# The Obesity-Fertility Protocol: a randomized controlled trial assessing clinical outcomes and costs of a transferable interdisciplinary lifestyle intervention, before and during pregnancy, in obese infertile women

**DOI:** 10.1186/s40608-015-0077-x

**Published:** 2015-12-01

**Authors:** Karine Duval, Marie-France Langlois, Belina Carranza-Mamane, Marie-Hélène Pesant, Marie-France Hivert, Thomas G. Poder, Hélène B. Lavoie, Youssef Ainmelk, Denise St-Cyr Tribble, Sheila Laredo, Ellen Greenblatt, Margaret Sagle, Guy Waddell, Serge Belisle, Daniel Riverin, Farrah Jean-Denis, Matea Belan, Jean-Patrice Baillargeon

**Affiliations:** Research Center of the Centre Hospitalier Universitaire de Sherbrooke, Sherbrooke, Québec Canada; Division of Endocrinology, Department of medicine, Faculty of Medicine and Health Sciences, Université de Sherbrooke, 3001-12e Avenue Nord, Sherbrooke, Québec J1H 5N4 Canada; Department of Obstetrics and Gynecology, Université de Sherbrooke, Sherbrooke, Québec Canada; Procrea Cliniques, Montréal, Québec Canada; Department of Economics, Faculty of Administration, Université de Sherbrooke, Sherbrooke, Québec Canada; UETMIS, Centre Hospitalier Universitaire de Sherbrooke, Sherbrooke, Québec Canada; Department of Medicine, Division of Endocrinology, Université de Montréal, Montréal, Québec Canada; Nursing School, Université de Sherbrooke, Sherbrooke, Québec Canada; Department of Medicine, Division of Endocrinology, Women’s College Hospital, University of Toronto, Toronto, Ontario Canada; Centre for Fertility and Reproductive Health, Mount Sinai Hospital, University of Toronto, Toronto, Ontario Canada; Department of Obstetrics and Gynaecology, University of Alberta, Edmonton, Ontario Canada; Department of Obstetrics & Gynecology, Université de Montréal, Montréal, Québec Canada; Quebec Ministry of Health, Québec, Québec Canada

**Keywords:** Obesity, Women, Fertility, Weight loss, Lifestyle, Pregnancy, Randomized controlled trial, Intervention, Polycystic ovary syndrome

## Abstract

**Background:**

Obesity in infertile women increases the costs of fertility treatments, reduces their effectiveness and increases significantly the risks of many complications of pregnancy and for the newborn. Studies suggest that even a modest loss of 5–10 % of body weight can restore ovulation. However, there are gaps in knowledge regarding the benefits and cost-effectiveness of a lifestyle modification program targeting obese infertile women and integrated into the fertility clinics. This study will evaluate clinical outcomes and costs of a transferable interdisciplinary lifestyle intervention, before and during pregnancy, in obese infertile women. We hypothesize that the intervention will: 1) improve fertility, efficacy of fertility treatments, and health of mothers and their children; and 2) reduce the cost per live birth, including costs of fertility treatments and pregnancy outcomes.

**Methods/Design:**

Obese infertile women (age: 18–40 years; BMI ≥30 kg/m^2^ or ≥27 kg/m^2^ with polycystic ovary syndrome) will be randomised to either a lifestyle intervention followed by standard fertility treatments after 6 months if no conception has been achieved (intervention group) or standard fertility treatments only (control group). The intervention and/or follow-up will last for a maximum of 18 months or up to the end of pregnancy. Evaluation visits will be planned every 6 months where different outcome measures will be assessed. The primary outcome will be live-birth rates at 18 months. The secondary outcomes will be sub-divided into four categories: lifestyle and anthropometric, fertility, pregnancy complications, and neonatal outcomes. Outcomes and costs will be also compared to similar women seen in three fertility clinics across Canada. Qualitative data will also be collected from both professionals and obese infertile women.

**Discussion:**

This study will generate new knowledge about the implementation, impacts and costs of a lifestyle management program in obese infertile women. This information will be relevant for decision-makers and health care professionals, and should be generalizable to North American fertility clinics.

**Trial registration:**

ClinicalTrials.gov NCT01483612. Registered 25 November 2011.

## Background

### Obesity and fertility

Infertility affects approximately 10–15 % of couples [1]. One of the leading causes in women is anovulation caused by the polycystic ovary syndrome (PCOS) [2, 3], which affects 6–10 % of women of childbearing age [4]. PCOS accounts for more than 70 % of anovulatory infertility in women [5]. This syndrome is defined by hyperandrogenism and anovulation/polycystic ovaries [3], and its etiology is related to elevated insulin resistance and obesity in genetically predisposed women [6–9]. Accordingly, 50–75 % of women with PCOS are obese in North America [10]. Obesity increases the risk of PCOS, but it has also been associated with reduced fertility even in ovulatory women [11–13]. As of 2008, 44 % of Canadian women of reproductive age (20 to 44 years) were reported as being overweight (body mass index (BMI) 25.0 to 29.9 kg/m^2^, 25 %) or obese (BMI ≥30.0 kg/m^2^, 19 %) [14]. Also, a survey in Scotland found a significantly higher proportion of obesity in infertile women [15].

Obesity increases the costs of fertility treatments and reduces their effectiveness. Indeed, pharmacological induction of ovulation is less effective in obese PCOS women [16], with the ovulatory dose of clomiphene being positively correlated with BMI [17]. Obesity delays time to conception when using intrauterine donor insemination [18] and reduces conception following assisted reproductive technologies (ART) [19], with higher required doses of gonadotropins [20], lower implantation and pregnancy rates [20–22], and higher miscarriage rates [20–23]. In addition to fertility disorders, obesity increases significantly the risks of many complications of pregnancy, such as gestational diabetes, pre-eclampsia, caesarean section, intrauterine death, which are further increased in morbidly obese women [24–26]. During the postpartum period, overweight mothers are more likely to have hypertension and thromboembolism, leading to a higher risk of maternal mortality [27]. Obesity has also been associated with an increased risk of birth defects, such as ventral wall, neural tube, cardiac and multiple congenital defects [28]. Pregnancy and delivery procedures in obese women are therefore more costly, mainly because of increased caesarean section rates, length of hospital stay and admission to neonatal intensive care [29–31]. It was estimated that the overall costs of fertility treatments and pregnancy complications were increased by 44–54 % and 70–100 % in overweight and obese women, respectively, compared to their normal weight counterparts [32]. Moreover, offspring from obese women are more likely to develop obesity, type 2 diabetes (DM2) and cardiovascular diseases in their lifetime, often at a younger age [33]. Studies suggest that maternal obesity, *via* a pro-inflammatory milieu, insulin resistance, or other hormonal factors [33, 34], modify the in utero environment and might induce fetal epigenetic programming [35] which predisposes to the development of obesity and DM2 early in life, thus perpetuating the vicious circle of obesity and its comorbidities in subsequent generations.

### Obesity management in women seeking fertility treatment

Collectively these substantive maternal and fetal risks call for women to lose weight prior to assisted conception in order to improve their fertility and chance of a healthy pregnancy [22, 36]. Indeed, it has been shown that a supervised lifestyle modification program, including support in addition to diet and exercise advice, can reduce weight, increase ovulatory frequency and improve pregnancy rates [37–39]. Even a modest weight loss of 5–10 % of total body weight can restore ovulation [40, 41]. The Fertility Fitness Program founded by Dr Norman in Australia is a 6-month program of weekly group sessions targeting behavioural changes that include exercise and diet. Clark et al. [39] found that in overweight, infertile, anovulatory women, this program resulted in an average weight loss of 6.3 kg, a restoration of ovulation in 12 of the 13 subjects and pregnancy in 11 women. In a follow-up study, 67 anovulatory obese women who completed the program lost an average of 10.2 kg with 90 % of them resuming spontaneous ovulation [38]. Of these participants, 78 % achieved a pregnancy and 67 % a live birth [38]. Recently, a randomized controlled trial (RCT) was completed in Australia with 171 obese infertile women and demonstrated that the program induced greater weight loss than the control intervention (4.7 vs. 1.3 kg, *P* < 0.001) and increased pregnancy rates at 18 months from 21 to 61 % (*P* < 0.001) (Clark et al., unpublished).

A study conducted by our group shows that it is possible for obese women with PCOS to achieve clinically significant and sustained weight loss by following simple advice given in a regular clinical care setting [42]. We retrospectively reviewed weight changes of young obese PCOS women and found that 43 % of 74 women lost ≥5 % of their initial weight after 6–12 months of follow-up. Young obese PCOS women seem to be a responsive population to lifestyle modification and it is thus expected that an integrated interdisciplinary lifestyle program will be even more effective in a similarly young population of obese women who are also highly motivated by their desire for a healthy baby.

Effective and acceptable lifestyle-modification strategies have the highest potential to result in a reduction in the socioeconomic burden of the obese pregnant woman. Indeed, the American Society for Reproductive Medicine [43], the American Dietetic Association [44], the American Society for Nutrition [44], the European Society of Human Reproduction and Embryology [45] and the British Fertility Society [46] have recommended that overweight/obese women should be provided with assistance to lose weight prior to conception and maintain a healthy lifestyle to prevent excess weight gain during pregnancy. The “Canadian clinical practice guidelines on the management and prevention of obesity in adults and children” emphasizes the importance of the multidisciplinary health care team for effective weight management [47]. After assessment of co-morbidities and readiness to change, a lifestyle modification program is proposed as the first step with an objective of 5–10 % weight loss. Our group implemented an interdisciplinary approach to obesity management at the *Centre hospitalier universitaire de Sherbrooke* (CHUS) since 2001. We have demonstrated in the general obese population that this low-cost approach leads to clinically significant weight loss [48–50]. However, access to our specialized programs is limited by a long waiting list with prohibitive delays for obese women seeking fertility treatment. They require rapid access to a lifestyle program tailored to their specific needs. At this time, there is no integrated lifestyle modification program targeting this specific population in the North American public health care systems. There are gaps in knowledge regarding the benefits and cost-effectiveness of such intervention in the North American context that our study will address. The development of a new initiative aiming at increasing efficiency and reducing the costs of fertility treatments, while improving the health of mothers and their offspring is needed.

### Hypothesis

We hypothesize that an interdisciplinary lifestyle intervention taking place before and during pregnancy in obese infertile women will: 1) improve fertility, efficacy of fertility treatments, and health of mothers and their children; and 2) reduce the cost per live birth and costs of fertility treatments and rates of adverse pregnancy outcomes. We further propose that our research program will 3) result in generalizable and transferable new knowledge useful for decision-makers of the health care system.

### Study objectives

This study protocol aims to design, implement and evaluate a transferable cost-effective program for lifestyle management of obesity in infertile women integrated into the fertility clinics. To achieve this goal, the proposed project will focus on four specific objectives:

1. To design and implement an interdisciplinary program for lifestyle management of obese women, or overweight women with PCOS, who seek fertility treatment at the CHUS, a fertility clinic with provincial coverage of fertility treatments and satellite access to *in vitro* fertilization (IVF);
2. To evaluate lifestyle benefits of this program and assess its impact on fertility, pregnancy complications and neonatal outcomes, as compared to a randomly assigned control group and to similar women seen in three fertility clinics across Canada with and without public coverage of fertility treatments;
3. To assess cost per live birth, and other measures of cost-effectiveness, of this program compared to the control group and three fertility clinics across Canada with and without public coverage of fertility treatments; and
4. To effectively transfer knowledge obtained through these activities to relevant stakeholders in the health care and public health sectors, including policy-makers, decision-makers, health care professionals and patients.

## Methods/Design

### Study design and setting

The proposed study relies on a participatory research approach which will combine quantitative and qualitative assessments. The impacts of the lifestyle program that will be developed will be assessed using a randomized controlled trial in one academic center. The study was registered to ClinicalTrials.gov (NCT01483612).

The RCT will be conducted at the CHUS fertility clinic, a fertility clinic with satellite access to IVF. The clinic offers care to patients with a range of reproductive problems including infertility, recurrent pregnancy loss, anovulation, PCOS, hyperprolactinemia, endometriosis, etc. The clinic has a successful ovulation induction and superovulation program including intrauterine insemination and access to donor sperm. Since 2011, it offers an IVF program in collaboration with Procrea Cliniques initially, and subsequently with the assisted reproduction center of the CHU Ste-Justine, where only the egg retrievals and embryo transfers are performed (evaluation of patients, protocol prescription, monitoring and decision making before and during cycles is done in Sherbrooke). Clinical outcomes and costs will be compared to a randomly assigned control group and to similar women seen in three fertility clinics across Canada not offering specific weight loss interdisciplinary lifestyle program: 1) an intra-provincial clinic (Procrea Cliniques, Montreal) with public coverage of fertility treatments; 2) 2 extra-provincial clinics (Centre for Fertility and Reproductive Health and IVF Unit at Mount Sinai Hospital, Toronto; and Fertility Clinic of Royal Alexandra Hospital, Edmonton) without public coverage of fertility treatments. These centers offer the full range of ART on site.

### Study participants

Study participants will be recruited among all women consulting at the CHUS fertility clinic. The staff will assist the research team in identifying potential participants. To be included in the study, women must fulfill the following inclusion criteria: 1) infertility; 2) aged between 18 and 40 years; and 3) obesity (BMI ≥30 kg/m^2^) or overweight if PCOS (BMI ≥27 kg/m^2^). Women will be excluded if they had bariatric surgery or plan for it and/or if natural conception is impossible or highly unlikely (tubal factor, severe male factor infertility, etc.). Infertility will be defined as: 1) failure to achieve a clinical pregnancy after 12 months or more of regular unprotected sexual intercourse in women who are under 35 years with regular menstrual cycles; 2) women who do not have regular menstrual cycles, or are older than 35 years and have not conceived during a 6-month trial period; or 3) women with a known cause of infertility. Eligible patients will be referred to the research coordinator to obtain their individual informed consent prior to baseline data collection.

### Ethical considerations

The proposed research was reviewed and approved by the Institutional Research Ethics Review Boards of the CHUS and other fertility clinics. Both research participants and health professionals will be duly informed and consent will be obtained in writing prior to evaluations or focus groups. This includes the 20 patients who will be randomly selected from other Canadian fertility clinics to be evaluated at the end of the study using the same questionnaires and focus group as patients at the CHUS. The main ethical consideration of the proposed research is confidentiality issues that will be minimized. All data will be coded, archived for at least 5 years and then destroyed.

### Randomisation

An independent statistician will use computerised random number generation to allocate each participant to the intervention or control group. Randomisation will be stratified according to the PCOS or non-PCOS status, based on clinical diagnosis in the patient’s record. Sequences will be generated using permuted block randomisation, with block sizes of two, four or six entries. The allocation sequence will be concealed in corresponding sequentially numbered opaque envelopes. After a participant has completed baseline assessments, the research coordinator will open the envelope to reveal the group allocation to the participant. Participants will be informed of their group assignment at that time.

### Intervention

#### Control arm

Participants assigned to the control group will receive standard fertility treatments, which will be initiated as soon as clinically indicated. Standard fertility treatments may include lifestyle counselling by the obstetrician-gynecologist certified in gynecologic reproductive endocrinology and infertility (GREI), reproductive endocrinologist or fertility specialist in charge of the care of the patient.

#### Intervention arm

Participants allocated to the intervention group will be invited to participate in the interdisciplinary lifestyle intervention and will have to delay other fertility treatments for six months. After 6 months of lifestyle intervention alone, women who are not pregnant will begin pharmacologic fertility treatments, as indicated, in conjunction with the lifestyle intervention that will continue to be offered. The aim of the intervention will be to implement progressive and sustainable lifestyle changes in order to reach a modest weight loss. Women will be invited to individually meet with a dietitian and a kinesiologist (20–30 min each) at 0, 3, 6 weeks and then every 6 weeks over a period of 18 months or until the end of pregnancy. A follow-up phone call/email will also take place once between each meeting for additional support. The dietitian will use 3-day food records to evaluate the women’s food intake throughout the program. To help improve patient’s diet, Canada’s Food Guide and the “Healthy Plate” [51] will be the main tools use for nutritional counselling. The kinesiologist will be responsible for taking anthropometric measures and vital signs and for coaching women to increase their physical activity level. A pedometer will be offered to assist the patient. Patients will be guided by the dietitian and the kinesiologist to formulate S.M.A.R.T goals (specific, measurable, attainable, realistic, and timely) at each meeting. The members of our interdisciplinary team will be trained in motivational interviewing. This training can positively affect the attitude to behaviour change in obese infertile women [52].

Besides individual intervention, women will benefit from a series of 12 educational group sessions (45-minute interactive small group workshops and 45-minute physical activity) covering various topics relevant to obesity management and fertility (Table 1). The workshops will be conducted by either the dietitian or psychologist and the demonstration of physical activity will be performed by the kinesiologist. These group sessions will take place weekly. When a pregnancy is confirmed, the woman will be met to set new objectives specific to her pregnancy, including an optimal gestational weight gain based on Institute of Medicine guidelines [53]. Partners will be invited to join individual meetings and strongly encouraged to assist all group sessions. To our knowledge, this is the first study assessing a lifestyle intervention in obese infertile women that is continued during fertility treatments and pregnancy.

**Table 1:** Topics of the interactive workshops and physical activities of the Obesity-Fertility group sessions program

Sessions	Interactive workshops	Physical activities
1	*Nutrition:* Canada’s Food Guide	Walking
2	*Psychology:* Couple Communication	Aquafitness
3	*Nutrition:* Energy Density of Foods: Healthy Substitutions for Weight Loss	Step Aerobics
4	*Psychology:* Stages of Behaviour Change and Motivation	Strength Training at Home
5	*Nutrition:* Environmental Factors – Impact on Food Intake	Interval Walking
6	*Psychology:* Sleep and Relaxation	Yoga
7	*Nutrition:* Managing Hunger and Satiety	Exercising While Watching TV
8	*Psychology:* Eating Disorders vs. Compulsive Overeating and Actions Strategies	Aquafitness
9	*Nutrition:* Meal Planning Tips/ Alcohol and Smoking	Walking in Combination with Cardio and Muscular Endurance Exercises
10	*Psychology:* Self-Esteem and Body Image	Strength Training with Exercise Ball and Resistance Band
11	*Nutrition:* Understanding a Food Label	Circuit Training
12	*Psychology:* Behaviour Self-Assessment	Aerobic Dance Workout

### Committees overseeing the study

To facilitate the inclusion of all relevant stakeholders, four committees will be involved throughout program development and implementation, as well as the evaluation process.

#### Steering committee

This committee will bring together relevant medical experts of various disciplines (endocrinology, obesity, GREI) and health care decision-makers from the CHUS. It will be responsible for supervising the development of the intervention, the planning of the evaluation, and the monitoring of the intervention implementation at the CHUS. It will overlook the work of all subcommittees and the course of implementation, with difficulties and proposed solutions, and will decide the directions for the intervention and the research plan. Steering committee meetings will occur regularly, as needed, and will also serve as knowledge transfer activities where decision-makers and knowledge users will be informed about the implementation and the results of the research.

#### Advisory committee

This committee will gather experts and decision-makers, who actively collaborate to this research project but are external to our institution, especially colleagues at the three fertility clinics of comparison. This committee will advise the research team on design of the intervention and planning of the evaluation, including evaluations on their sites. The Advisory committee will meet as needed and will be actively involved in revising documents and through informal communications. Post-intervention meetings will also serve as knowledge transfer activities.

#### Intervention sub-committee

This sub-committee will be in charge of developing the intervention in all its details and to follow-up closely on its implementation. This sub-committee will meet every month before the project begins and then as needed until the end of the intervention.

#### Evaluation sub-committee

This sub-committee will develop all the evaluation tools and will plan the qualitative evaluations. It will be particularly active before the start of the intervention and at the end of the intervention for the qualitative evaluations on all sites.

### Development & implementation of the lifestyle program for obese infertile women (Objective 1)

The Intervention sub-committee will be directly in charge of defining the intervention and planning the implementation. A complete review of the relevant literature will be performed initially and made available to all other committee members. The implementation of the program will be closely monitored by this sub-committee with evaluation of required human, material and other resources; barriers that will be encountered; potential solutions that will be applied; and discussion of difficult cases managed in the program. In addition, a specific qualitative evaluation of the program implementation will take place at the end of the intervention and will be planned by the Evaluation sub-committee. All women included in the CHUS RCT will be evaluated by questionnaires and a sub-group of 20 of these patients will be selected to participate in focus groups. In order to compare health professionals’ and patients’ satisfaction, perceptions and impact of the program on their life to those during usual care in Canada, we recruited three Canadian fertility clinics with different characteristics. In these clinics, 20 patients randomly selected will be evaluated at the end of the study using the same questionnaires and focus group methods. In all four centers, health care providers involved in the fertility clinic and/or the program will also be evaluated by questionnaires and focus groups.

This objective will be under the supervision of our Steering committee and Advisory committee.

Decision-makers from Quebec and Canada who sit on these committees will ensure that our program meets their objectives and is acceptable in terms of required resources and costs. Furthermore, we will validate our program design and evaluation plan with worldwide key experts as well as Canadian obesity experts contacted through the Canadian Obesity Network (CON). We will benefit from the experience of experts involved in the implementation and evaluation of similar programs: the Fertility Fitness program in Australia and the Lausanne Obesity-Fertility clinic in Switzerland.

### Evaluation of clinical outcomes of the lifestyle program for obese infertile women (Objective 2)

The **primary outcome** will be live-birth rates at 18 months. **Secondary outcomes** will be sub-divided into four categories of clinical outcomes:

1. ***Lifestyle and anthropometric outcomes***: evolution of anthropometric measures, changes in lifestyle habits (diet, exercise, sleep, alcohol, and tobacco), physical fitness level, daily energy expenditure from physical activities, time spent in various physical activity intensities, step count, evolution of readiness for change, quality of life.
2. ***Fertility outcomes***: pregnancy rate (biochemical pregnancy confirmed by a positive beta-human chorionic gonadotropin (β-hCG) level in the serum), spontaneous abortions, multiple pregnancies, spontaneous vs. ART-induced pregnancy, doses of fertility medications, number of treatment or IVF cycles.
3. ***Pregnancy adverse outcomes***: gestational diabetes, hypertension, pre-eclampsia, thromboembolism, caesarean section.
4. ***Neonatal outcomes***: birth weight, hypoglycemic episodes, Apgar score, jaundice, admission to neonatal intensive care, congenital defects, intrauterine death.

The results obtained at the CHUS will also be compared to those of women with the same criteria who will be seen in three fertility clinics across Canada. At the CHUS, primary and secondary outcomes will be determined using patients’ assessments, questionnaires and review of patients’ records. At the comparative clinics, charts of patients seen during the same time period will be retrospectively reviewed, and administrative data will be gathered.

### Cost-effectiveness assessment of the lifestyle program for obese infertile women (Objective 3)

Data collected for this objective will trace the operating costs (human and material resources) for each of the patients in the study based on two viewpoints: 1) the costs for the public health system; and 2) the costs for the patients. The two viewpoints will translate into two models of costs for all analyses. The key variable of interest is the total cost per live birth. This variable will be measured in two ways: 1) the total cost per infant alive for all women, which considers costs of unsuccessful interventions; and 2) the total cost per infant alive only for the women who had a live birth. Costs will include:

#### Costs of the lifestyle program

*Health care system viewpoint* – Costs for health care providers and staff (medical visits with corresponding billing, number of hours worked by health professionals and administrative staff), interdisciplinary meetings with case discussion, meetings related to the operation of the program, medical testing, consumables. *Patients’ viewpoint* – Costs for transportation, parking, loss of salary (patient & life partner), daycare.

#### Costs of fertility treatments

*Health care system viewpoint –* Costs for health care providers and staff (idem), drugs, medical testing, consumables, and billing of ART-related investigations or treatments to the public system if applicable. *Patients’ viewpoint* – Costs for transportation, parking, loss of salary, daycare, drugs not covered, and billing of ART-related investigations or treatments to the patient if applicable.

#### Costs of adverse events or complications

*Health care system viewpoint* – Costs of hospitalization or drugs for adverse events related to the lifestyle program, fertility treatments, pregnancy or neonatal care. *Patients’ viewpoint* – Costs for transportation, parking, loss of salary, daycare, drugs not covered, incurred by the patients due to these adverse events or complications.

Data collection will be performed through patients’ charts review, administrative data, questionnaires and interviews with health care providers and staff of fertility clinics for the description of care procedures, as well as questionnaires to patients. At the CHUS fertility clinic, costs will be obtained prospectively and all patients will answer a specific questionnaire; but for the three fertility clinics of comparison, patients’ charts will be retrospectively reviewed. However, as mentioned, a sample of 20 patients will be selected for focus groups: these patients will also answer the same cost questionnaire. Thus, the exact costs from the patients’ viewpoint of the different events will be determined based on these 20 patients and then be extrapolated to the other patients. Departments or directorates of service (billing services at the CHUS, pharmacies, Human Resources, Department of Nursing) will be consulted to match each event to its monetary cost. Where possible, we will use the overhead costs, provided by the administrative services, for a particular treatment and/or complication. When this is not possible, we will conduct interviews with relevant professionals to track the various human resources and materials that were involved.

Since provincial and state pricings for the different outcomes are different, we will only consider a single reference price for each outcome, i.e. pricing in the Quebec health system. In order not to incur double counting in calculating the cost of primary outcome, we will make sure to check the reason for the expense and we will record every cost only once for the primary outcome. Regarding the joint use of equipment or spaces, the cost used will be the unit cost of depreciation for each use or visit. To the extent that the cost measure will be done over a period exceeding one year, we will use a discount rate. The reference discount rate will be 5 % per year. To test the robustness of our results, we also do a sensitivity analysis with rates of 3 and 8 %.

Assessment of quality-adjusted life years (QALYs) and cost-effectiveness of the intervention will be performed using the Short Form-6D (SF-6D). SF-6D is a generic preference-based measure of health derived from the SF-36 Health Survey for use in economic evaluation [54]. It is composed of six dimensions of health: physical functioning, role limitations, social functioning, pain, mental health and vitality. Each dimension has between 4 and 6 levels. The SF-6D defines 18,000 different possible health states, and has a range from 0.3 (worst health state) to 1.0 (best health state) in model 10 [54].

### Variables and research tools

Specific research tools for each objective are summarized in Table 2, which includes corresponding variables as well as the sources that will be used to obtain required data and the time of data collection.

**Table 2:** Summary of methodology and approach

Variables	Sources	Time of data collection	
		CHUS-RCT	Comparison fertility clinics
1. Development and implementation of the interdisciplinary lifestyle intervention			
Health professionals’ perceptions and satisfaction toward obesity and fertility management	Questionnaire	At the end of the study	Retrospectively
	Focus groups		
Patients’ perceptions and satisfaction toward weight management and fertility care	Questionnaire	At 18 months or 24–28 weeks pregnant	At the end of the study
	Focus groups		
2. Evaluation of lifestyle benefits and impact on fertility, pregnancy complications and neonatal outcomes			
Live-birth rate	Review of patient medical record	Throughout the study	Retrospectively
Lifestyle and anthropometric outcomes^a^		Baseline, 6 months, 12 months, and 18 months. Among women who become pregnant: beginning of the pregnancy and 24–28 weeks pregnant.	N/A
∙ *Anthropometric measures*	Standard calibrated scale and standing electric bioimpedance		
∙ *Vital signs*	Automatic blood pressure monitor		
∙ *Metabolic markers related to insulin secretion, androgen, lipid profile and OGTT*	Blood sample		
∙*Lifestyle habits (diet, exercise, sleep, alcohol, and tobacco)*	Questionnaire adapted from the one used by Statistics Canada for the latest Canadian Health Survey		
∙ *Physical fitness level*	Six-minute walk test		
∙ *Daily energy expenditure from physical activities, time spent in various physical activity intensities and step count*	Tri-axial accelerometer		
∙ *Readiness for change*	Questionnaire (WLRT [75])		
∙ *Quality of life*	Questionnaire (FertiQoL [76])		
Fertility outcomes		Throughout the study	Retrospectively
∙ *Pregnancy rate*	Positive β-hCG level in the serum		
∙ *Others*	Review of patient medical record		
Pregnancy adverse outcomes	Review of patient medical record	Throughout the study	Retrospectively
Neonatal outcomes	Review of patient medical record	Throughout the study	Retrospectively
3. Evaluation of cost per live birth/cost-effectiveness of the interdisciplinary lifestyle intervention			
Costs of the interdisciplinary lifestyle intervention^a^	Questionnaires	Throughout the intervention	N/A
	Administrative data		
Costs of fertility treatments	Review of patient medical record	Throughout the study	Retrospectively
	Questionnaires		
	Administrative data		
Costs of adverse events or complications	Review of patient medical record	Throughout the study	Retrospectively
	Questionnaires		
	Administrative data		
4. Knowledge transfer			
Scientific meeting presentations	ASRM, CSEM, ENDO and other annual meetings	Throughout the study	
Publication in scientific journals	Human reproduction, Fertility and sterility, Journal of clinical endocrinology & metabolism, etc.	Throughout the study	
Presentations to decision-makers and knowledge users	Solicited and unsolicited invitations	Throughout the study	
Diffusion to stakeholders	Conduit, letters, e-mails	Throughout the study	
Executive summary		At the end of the study	

*ART* Assisted reproductive technologies, *β-hCG* Beta-human chorionic gonadotropin, *IVF In Vitro* Fertilization, *ASRM* American society for reproductive medicine, *CSEM* Canadian society of endocrinology and metabolism, *ENDO* Endocrine society, *FertiQoL* Fertility quality of life tool, *OGTT* Oral glucose tolerance test, *WLRT* Weight loss readiness tool

^a^CHUS-RCT: *Centre hospitalier universitaire de Sherbrooke*-randomized controlled trial

#### Health professionals’ perceptions and satisfaction (Objective 1)

Health professionals’ attitudes, perceptions, self-efficacy toward obesity management, and other characteristics (age, experience in obesity management) will be evaluated with a questionnaire that we have developed and used in previous studies [55]. Self-efficacy is defined as the set of beliefs about one’s capabilities to perform at a designated level. It is particularly important in the context of the implementation of a new program since it may be influenced by the practice environment [56]. For health professionals to engage in weight management with their patients, they must not only accept this as part of their role but also feel that they are competent to accomplish the task. Negative attitudes toward obese individuals and perceptions to the effect that available treatments are ineffective can prevent the engagement of health professionals in weight management. There is also a positive correlation between the feeling of competency and the degree of engagement regarding various aspects of obesity management and improved attitude towards obese individuals [57, 58]. A separate questionnaire will gather detailed information on strengths of the program, difficulties and areas of possible improvement.

A qualitative in-depth analysis of key stakeholder perceptions of the program and fertility clinics will be performed using semi-structured interviews (MDs, nurses, dietitians, kinesiologists, psychologists, clinic administrative personnel and directors), as we have previously done [59, 60]. This will increase our understanding of their personal experience, inter-professional collaboration and satisfaction with the program, and further identify and detail strengths and areas for potential improvement. This will also permit a better understanding of the change process.

#### Patients’ perceptions and satisfaction (Objective 1)

Patient expectations and perceptions of their personal experience regarding weight management, fertility care and pregnancy follow-up, as well as their satisfaction regarding management by their health professionals will be evaluated with questionnaires based on experience from previous studies. A qualitative in-depth analysis of patients’ perceptions of the program and medical care will also enhance our understanding of their personal experience with the program. After the completion of the quantitative evaluation of impact of the program on patients, a theoretical sample based on the technique of critical incidents will be used to create a sub-sample of the whole group. Twenty patients will be recruited in each of the 4 participating clinics (80 patients). To be recruited those patients will have to show certain characteristics (different levels of satisfaction with their care (at CHUS, based on questionnaires), different fertility or pregnancy outcomes, loss or gain of weight during follow-up, etc.). The patients will be participating in a taped-recorded semi-structured qualitative interview to assess their perceptions of impact of their participation into the program (continuity of care, perceptions of changes, contribution to change, quality of life, perspectives of future changes, explanation of level of satisfaction with the program). An interview guide with open-ended questions adapted from the principal variables of the study, the Diabetic Empowerment Scale (DES) [59], and the Impact of Weight on Quality of Life (IWQOL-lite) questionnaire [61] will be used as done in previous studies from members of our group [49, 62].

#### Evaluation of anthropometric and lifestyle benefits and impact on fertility, pregnancy and neonatal outcomes (CHUS only) (Objective 2)

**Fig. 1:**
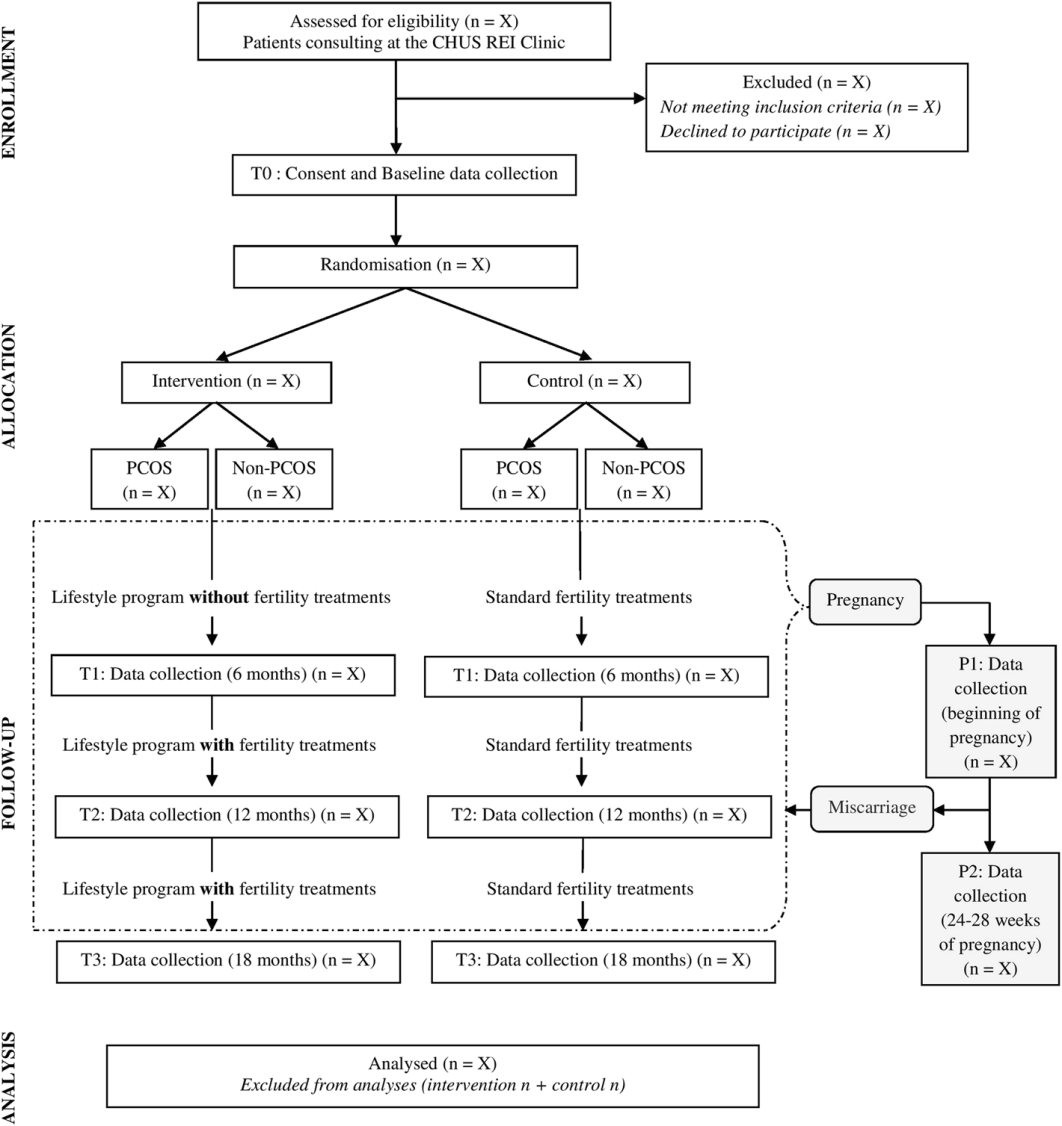
Summary of design and data collection of the Obesity-Fertility protocol

Evaluations of anthropometric measures and lifestyle habits will take place at baseline, prior to randomisation, and then every 6 months for 18 months. Among women who become pregnant, data will be collected, at the beginning of the pregnancy and at 24–28 weeks. If a woman becomes pregnant within a month of a standard evaluation visit, this visit will be considered as the first pregnancy visit. If a miscarriage occurs, the original follow-up will continue until 18 months or the end of another pregnancy. An overview of the study design and data collection is provided in Fig. 1.

##### Anthropometric measures and vital signs:

Weight will be measured by a standard calibrated scale and height will be measured with a stadiometer. In non-pregnant women, waist circumference will be measured with a measuring tape, as recommended [47, 63]. Fat mass and percent body fat will be measured by standing electric bioimpedance, which was shown to be reliable compared to underwater weighing and supine tetrapolar bioimpedance [64, 65]. Blood pressure and pulse rate will be measured after five minutes of rest, in the sitting position. The average of two measurements will be used for analysis.

##### Blood sample:

Different metabolic markers related to insulin secretion, androgen and lipid profile will be measured fasting: apolipoprotein B (ApoB), total cholesterol, triglycerides, high-density lipoprotein cholesterol (HDL), low-density lipoprotein cholesterol (LDL), cholesterol ratio, thyroid-stimulating hormone (TSH), prolactin (PRL), cortisol, adrenocorticotropic hormone (ACTH), β-hCG, total and free testosterone, Sex hormone-binding globulin (SHBG), 17-hydroxyprogesterone, dehydroepiandrosterone sulfate (DHEAS), androstenedione, C-peptide, glucose and insulin. A 2-hour oral glucose tolerance test (OGTT) will be also performed with blood sample taken every 30 min. Samples will be stored at each time points for future measurements of adipokines or other analytes pertinent to the objectives of this study, if funding allows it.

##### Lifestyle:

Physical activity level and eating habits will be measured using a questionnaire adapted from the one used by Statistics Canada for the latest Canadian Health Survey. This questionnaire inquires about the frequency and duration of active travelling, leisure time, and sedentary activities (television watching, video games, computers). We chose this questionnaire since it allows comparison of the characteristics of our patients to those of the general Canadian population. Moreover, women will be asked to wear a tri-axial accelerometer (GT3X+, Actigraph, Pensacola, FL, USA) on their hip for seven consecutive days except during sleep and showering/bathing. The accelerometer will be used to estimate daily energy expenditure from physical activities, time spent in various physical activity intensities and step count. The GT3X+ has been shown to be a valid and reliable measure of physical activity in free living conditions [66].

##### Fitness level:

The six-minute walk test (6MWT) is a simple test that has been used to estimate functional capacity in obese subjects and is also a predictor of morbidity and mortality [67–69]. The 6MWT will be performed according to the protocol recommended by the American Thoracic Society in a 20-meter hallway marked every 5 m with coloured tapes on the floor, and measures the distance that a patient can quickly walk in a period of 6 min [70]. It has been shown that weight loss increases fitness level early in the weight loss process, making this test a very accurate tool to evaluate the impact of the intervention on obese or overweight patients’ functional capacity [71–73].

##### Readiness for change:

In the course of previous prospective studies, we have designed a 22-question weight loss readiness tool (WLRT) questionnaire based on Prochaska and DiClemente’s Stages of Change Model [47, 74]. It evaluates the motivational readiness of patients regarding weight management, nutrition and physical activity, and could predict response to an intervention. Our WLRT takes 5–10 min to fill out, making it a clinically applicable tool. It has been useful to identify subjects with greater chances of success for lifestyle modification in a previous pilot study [75]. Its predictive value will be assessed in this study, but it will also serve to explore the mechanisms of potential benefits of our intervention and for stratified analyses.

##### Quality of life:

The impact of fertility problems and its treatment on quality of life will be measured using the fertility quality of life (FertiQoL) tool, a reliable and sensitive questionnaire to evaluate quality of life in individuals with fertility problems [76].

#### Evaluation of fertility, pregnancy complications and neonatal outcomes (all sites)

##### Medical records:

Chart review of the mother will be performed using a standardized evaluation grid, and we will check personal medical and surgical history, medications, weight, and any particular events at each visit. This chart review will also identify information about fertility treatments and pregnancy complications. Information about the newborn will be collected *via* the obstetric file of the mother.

##### Administrative data and other databases:

Data from the chart review will be cross-validated with administrative data and any databases used in fertility clinics and/or local hospitals. At the CHUS, all laboratory testing and all information regarding hospitalisations are merged together and kept in the “*Centre informatisé de recherche évaluative en services et soins de santé”* (CIRESSS) database, which is available for research with appropriate authorizations.

### Data analyses and interpretation

For objectives 1–2, all comparisons between randomized groups at the CHUS will follow both the intent-to-treat and/or per-protocol principles, and will consider results at the end of study. For repeated measures, the last-observation-carried-forward method will be used for women who drop out or become pregnant (depending on analyses). Time-to-event analyses will also be performed for fertility outcomes, with censoring for drop out alone, as well as for anthropometric outcomes, with censoring for both drop out and occurrence of pregnancy. Univariate analyses will compare the change in a variable in the intervention group with the change in this variable in the control group, using two-tailed unpaired *t* tests (for continuous variables) and chi-square or Fisher’s exact tests (for categorical variables). Changes in weight will also be compared for the same follow-up duration with changes for women seen at the other fertility clinics, using ANOVA tests. For the other variables, only end-of-study comparisons will be performed between the two groups in Sherbrooke and the three other fertility clinics, using ANOVA tests (for continuous variables) and logistic regressions (for categorical variables). Bonferroni-corrected p-values will be performed to adjust for the multiplicity of comparisons and an α level of 5 % will be considered significant for all analyses. Continuous variables that are not normally distributed will be log-transformed in order to ascertain normal distribution. Multivariate analyses will also be used in order to adjust for baseline differences and for potential confounders, and to determine independent predictors of outcomes. Triangulation will be performed between quantitative and qualitative data.

### Sample size

Our sample size estimation is based upon our primary outcome (see Objective 2), which is live-birth rates between randomized groups at the CHUS. Based on the Fertility Fitness Program, the inclusion of 58 women per group will provide our study a power of 80 % to identify a doubling in live-birth rates with our program (25 to 50 %, α = 5 %). Assuming a dropout rate of 10 % (these women are usually highly motivated), our final estimated sample size will be 128 women. For other objectives and outcomes, it would be difficult to assess power because similar evaluations have not been previously reported.

### Knowledge translation and transfer plan (Objective 4)

It is worth mentioning that both our research program and research design are optimal to allow knowledge translation (KT) and application throughout the entire research process. Integrated KT is an important outcome of this research and will take form of regular meetings of the Advisory and Steering committees. Because of the involvement on these committees of representatives of Quebec and Ontario Ministries of Health, fertility clinics and CON, as well as expert clinicians, diffusion of results and exchange of experiences will be facilitated.

However, research by itself does little to induce change (except for participants) and thus, results have to be diffused to interested audiences. Our research is relevant to health professionals, healthcare system decision-makers and policy-makers, patients and Canadian population. We will reach out to these target audiences through linkage and exchange activities, including presentation of study results at local, national and international scientific meetings attended by interested stakeholders in the obesity, reproductive endocrinology, fertility and health services fields and publication in scientific journals, but also by direct reports to healthcare system decision-makers. A clear summary of research results, including key messages targeted for each selected audience and synthesized results, will be available (printed and on a web site). Local and national media will be invited to press conferences and we will schedule private meetings with important decision-makers to whom our findings are relevant.

## Discussion

This paper has outlined the protocol of a study assessing clinical outcomes and costs of a transferable interdisciplinary lifestyle intervention, before and during pregnancy, in obese infertile women. We are using a RCT design for proximal clinical outcomes, and mixed design for cost-effectiveness evaluation and qualitative assessment for the purpose of future dissemination and knowledge transfer. To our knowledge, we are the first in the world to conduct such a program with lifestyle intervention continuing throughout fertility treatments and pregnancy. This project is very important as it will generate new knowledge about the implementation, impacts and costs of a lifestyle management program in obese infertile women. The results of this study will provide valuable information on feasibility and transferability of such a program, including identification of barriers and solutions. We expect that our program will improve the lifestyle and fertility of the participants and reduce maternal and fetal complications during pregnancy, as compared with our different control groups. A significant reduction in cost per live birth and cost-effectiveness ratio is also anticipated.

Although this research protocol is innovative and uses a robust methodology, some limitations need to be acknowledged. First, implementation and outcome evaluation of the lifestyle program will take place in a single clinic. However, a RCT at the CHUS will be performed with sufficient power to assess the primary outcome. Results will also be compared to three other clinics, which is an ideal first step for the future development of a multicenter RCT. It is important to appropriately design and evaluate the program in one center before transferring it to other centers. Second, some of the outcome measures will be self-reported and might be biased. However, such bias should be similar in both randomized groups and the results will be triangulated with data from many different sources (administrative data, questionnaires, focus groups, etc.). Third, most of the outcomes will be assessed only retrospectively in the clinics of comparison. This might introduce an evaluation bias although multiple sources of information will be used and compared. A recall bias is also possible for the evaluation of costs for the patients’ viewpoint in these clinics, but the same estimations will be performed in Sherbrooke and compared with real costs, which will allow the determination of the degree of this bias for comparison clinics. Fourth, in Sherbrooke, fertility treatments, including IVF, will be covered by the public health care system. Therefore, some results may not reflect all North American fertility clinics, but the same results will be compared to clinics in other provinces where fertility treatments are not covered.

The outcome measures of this study will be relevant to a number of health systems’ managers and policy-makers. Indeed, new approaches aiming at increasing efficiency and reducing costs of fertility treatments, while improving care, are thus very pertinent for these policy-makers. This research proposal is completely in line with those objectives and is developed in partnership with local, provincial and national decision-makers, which increases the potential impact and use of the findings. Generalizability of our findings is increased by the evaluation of clinics from three different provinces and the involvement of the CON as a partner. Furthermore, experts and decision-makers from various provinces will participate on the Advisory committee to ensure that the developed intervention can be applied in other contexts. Early and active involvement of decision-makers will ensure that the intervention is appropriately designed to be implemented in most North American fertility clinics.

## Abbreviations

6MWT: Six-minute walk test
ACTH: Adrenocorticotropic hormone
ApoB: Apolipoprotein B
ART: Assisted reproductive technologies
β-hCG: Beta-human chorionic gonadotropin
BMI: Body mass index
CIRESSS: *Centre informatisé de recherche évaluative en services et soins de santé*
CHUS: *Centre hospitalier universitaire de Sherbrooke*
CON: Canadian Obesity Network
DES: Diabetic Empowerment Scale
DHEAS: Dehydroepiandrosterone sulfate
DM2: Type 2 diabetes
FertiQol: Fertility quality of life
GREI: Gynecologic reproductive endocrinology and infertility
HDL: High-density lipoprotein cholesterol
IVF: *In vitro* fertilization
IWQOL-Lite: Impact of Weight on Quality of Life
KT: Knowledge translation
LDL: Low-density lipoprotein cholesterol
OGTT: Oral glucose tolerance test
PCOS: Polycystic ovary syndrome
PRL: Prolactin
QALYs: Quality-adjusted life years
RCT: Randomized controlled trial
SF-6D: Short Form-6D
SHBG: Sex hormone-binding globulin
TSH: Thyroid-stimulating hormone
WLRT: Weight loss readiness tool
